# [^18^F]FDG and [^18^F]NaF as PET markers of systemic atherosclerosis progression: A longitudinal descriptive imaging study in patients with type 2 diabetes mellitus

**DOI:** 10.1007/s12350-021-02781-w

**Published:** 2021-09-13

**Authors:** M. Reijrink, S. A. de Boer, C. A. te Velde-Keyzer, J. K. E. Sluiter, R. A. Pol, H. J. L. Heerspink, M. J. W. Greuter, J. L. Hillebrands, D. J. Mulder, R. H. J. A. Slart

**Affiliations:** 1 Div. Vascular Medicine, Dept. Internal Medicine, University Medical Center Groningen, University of Groningen, Groningen, The Netherlands; 2 Div. Pathology, Dept. Pathology and Medical Biology, University Medical Center Groningen, University of Groningen, Groningen, The Netherlands; 3 Div. Nephrology, Dept. Internal Medicine, University Medical Center Groningen, University of Groningen, Groningen, The Netherlands; 4 Department of Vascular and Transplant Surgery, University Medical Center Groningen, University of Groningen, Groningen, The Netherlands; 5 Dept. Clinical Pharmacy and Pharmacology, University Medical Center Groningen, University of Groningen, Groningen, The Netherlands; 6 Dept. of Radiology, University Medical Center Groningen, Medical Imaging Center, University of Groningen, Groningen, The Netherlands; 7 Biomedical Photonic Imaging, Faculty of Science and Technology, University of Twente, Enschede, The Netherlands; 8 Dept. Nuclear Medicine and Molecular Imaging, University Medical Center Groningen, University of Groningen, Groningen, The Netherlands

**Keywords:** Inflammation, diabetes, atherosclerosis, PET, CT, vascular imaging

## Abstract

**Background:**

While [^18^F]-fluordeoxyglucose ([^18^F]FDG) uptake is associated with arterial inflammation, [^18^F]-sodium fluoride ([^18^F]NaF) is a marker for arterial micro-calcification. We aimed to investigate the prospective correlation between both PET markers over time and whether they are prospectively ([^18^F]FDG) and retrospectively ([^18^F]NaF) related to progression of systemic arterial disease in a longitudinal study in patients with type 2 diabetes mellitus (T2DM).

**Methods:**

Baseline [^18^F]FDG PET/Low Dose (LD) Computed Tomography (CT) scans of ten patients with early T2DM without cardiovascular history (70% men, median age 63 years) were compared with five-year follow-up [^18^F]NaF/LDCT scans. Systemic activity was expressed as mean target-to-background ratio (_mean_TBR) by dividing the maximal standardized uptake value (SUV_max_) of ten arteries by SUV_mean_ of the caval vein. CT-assessed macro-calcifications were scored visually and expressed as calcified plaque (CP) score. Arterial stiffness was assessed with carotid-femoral pulse wave velocity (PWV). Five-year changes were expressed absolutely with delta (Δ) and relatively with %change.

**Results:**

Baseline _mean_TBR[^18^F]FDG was strongly correlated with five-year follow-up _mean_TBR[^18^F]NaF (*r* = 0.709, *P* = .022). _mean_TBR[^18^F]NaF correlated positively with ΔCPscore, CPscore at baseline, and follow-up (*r* = 0.845, *P* = .002 and *r* = 0.855, *P* = .002, respectively), but not with %change in CPscore and PWV.

**Conclusion:**

This proof-of-concept study demonstrated that systemic arterial inflammation is an important pathogenetic factor in systemic arterial micro-calcification development.

**Supplementary Information:**

The online version contains supplementary material available at 10.1007/s12350-021-02781-w.

## Background

Inflammation and progressive calcification are the hallmarks of atherosclerosis, resulting in arterial stiffness and development of cardiovascular diseases.^1^,^2^ Although these processes are closely related, clinical data on their association over time are limited. With positron emission tomography (PET), detailed molecular imaging of the active processes of atherosclerosis can be visualized.^3^ [^18^F]-fluorodeoxyglucose ([^18^F]FDG) and [^18^F]-sodium fluoride ([^18^F]NaF) PET scans are suitable for assessment of respectively arterial inflammation and micro-calcification, and are proven useful for detection of cardiovascular disease in the clinical research setting.^3^ Arterial [^18^F]FDG uptake is linked to Pulse Wave Velocity (PWV)-assessed arterial stiffness and has shown to predict future cardiovascular events.^4^,^5^ Furthermore, [^18^F]FDG may also serve as a marker for endothelial dysfunction and as a precursor of arterial calcification, which are important characteristics of the development of cardiovascular disease. Patients with T2DM are at increased cardiovascular risk, with increased arterial inflammation on [^18^F]FDG PET and more progressive arterial calcification.^6^ Ex vivo studies suggest a close relation between PET-assessed arterial inflammation and calcification, but this has yet to be confirmed in clinical studies.^7^,^8^ Importantly, in a cross-sectional study, focal arterial [^18^F]FDG uptake did not correlate with [^18^F]NaF uptake.^9^ Furthermore, arterial [^18^F]FDG PET uptake was not associated with calcification progression on computed tomography (CT) and specific sites of increased [^18^F]FDG uptake and calcification rarely overlapped.^10^ However, since inflammation is the precursor of calcification development and previous studies observed that both processes are not present simultaneously,^9^,^11^ we hypothesize that [^18^F]FDG-assessed arterial inflammation may be associated with [^18^F]NaF-assessed arterial micro-calcification over time. The primary aim of this proof-of-concept study is the assessment of the prospective association of baseline systemic arterial [^18^F]FDG activity with systemic [^18^F]NaF PET during five years of follow-up in asymptomatic patients with T2DM. The secondary aim was to correlate arterial PET activity with CT-assessed macrocalcification and arterial stiffness and changes in these markers over five years.

## Methods and Results

### Study Design and Population

For this study, ten participants from the RELEASE trial were included (NCT02015299).^5^,^12^ The study design and selection of participants have been reported previously.^5^ In short, patients with early T2DM, without using glucose lowering drugs, and without severe cardiovascular history (i.e., stable coronary artery disease or acute coronary syndrome, stroke, or transient ischemic attack, peripheral artery disease) were included. Baseline assessments (including [^18^F]FDG PET/CT scan, clinical, laboratory, and cardiovascular assessments), were conducted between April 2014 and April 2015 and the follow-up visit (including [^18^F]NaF PET/CT scan, clinical, laboratory, and cardiovascular assessments) took place between January 2019 and June 2019. Both studies (baseline and follow-up) were reviewed and approved by the Medical Ethical Institutional Review Board of the UMCG (METC numbers 2013-080 and 2018-456, respectively). Both studies were performed in compliance with the principles of the Declaration of Helsinki.

### Clinical and Laboratory Assessments

At baseline and follow-up visit and a detailed medical history were evaluated. Height and weight were measured to determine body mass index (BMI). Blood samples were obtained in the morning after at least 8 hour of overnight fasting for the measurements of plasma glucose, HbA1_c_, and lipid profile. To determine arterial stiffness, carotid-femoral pulse wave velocity (PWV) and blood pressure measurements were performed at baseline and follow-up visit. Measurements were performed as described previously.^5^

### PET/CT Imaging

**Figure 1:**
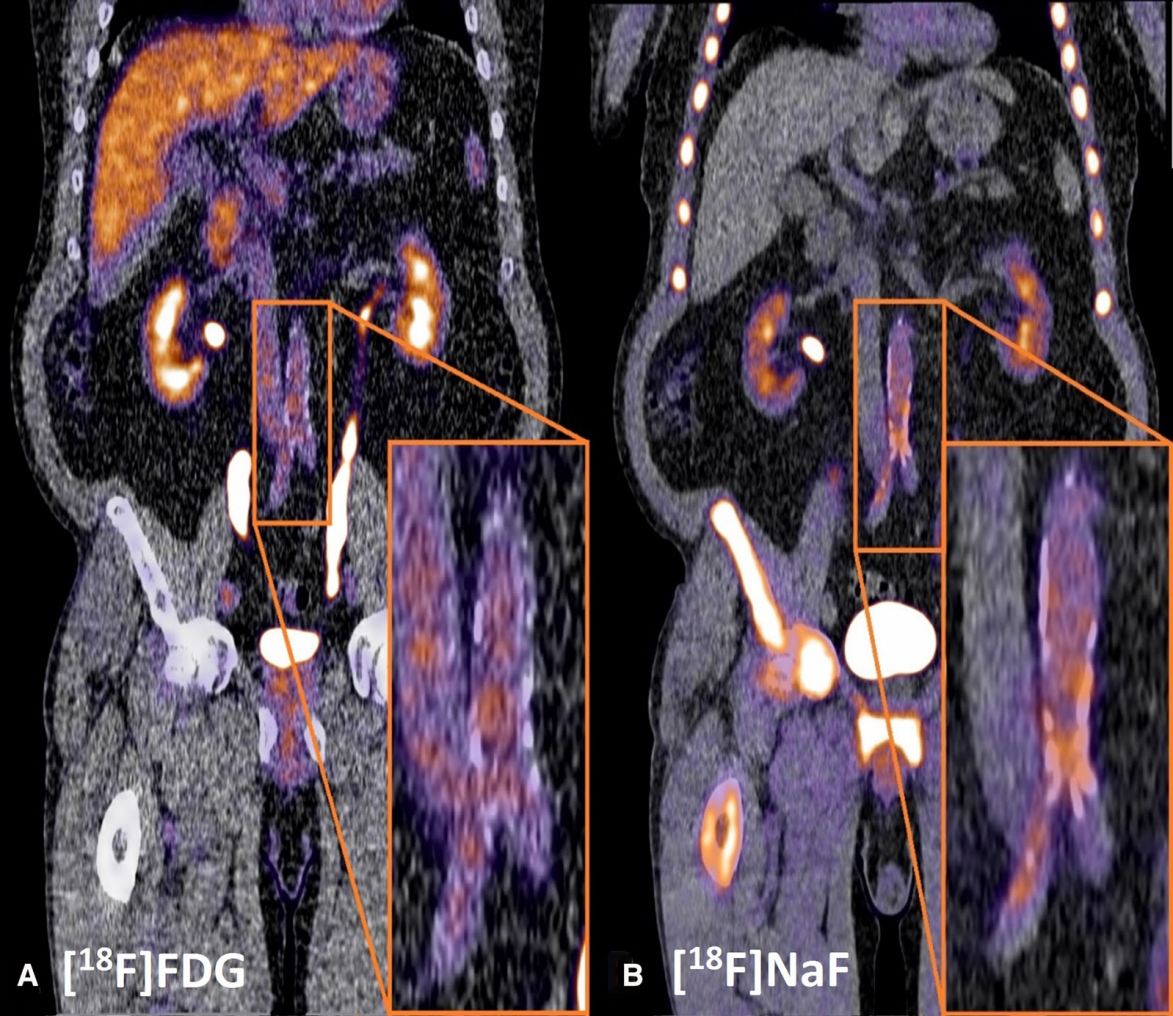
[^18^Fluor]fluordeoxyglucose ([^18^F]FDG) and five-year follow-up [^18^Fluor]SodiumFluoride ([^18^F]NaF) positron emission tomography/low dose computed tomography in a patient with type 2 diabetes mellitus. **A** Metabolic active cells are visualized by [^18^F]FDG uptake can be found in the liver, heart, and more focal in arterial tissue (pop-up). In arterial tissue, [^18^F]FDG uptake is a marker for inflammation which is considered an important factor in the development of CVD. **B** [^18^F]NaF uptake demonstrates skeletal activity and, next to that, this tracer also visualizes micro-calcification formation in the arterial wall (pop-up). Both nuclear tracers are excreted via the kidneys, ureters, and bladder. For image processing and analysis Affinity 2.0 was used (Hermes Medical Solutions)

At baseline, [^18^F]FDG PET/Low Dose (LD) CT imaging was performed on a Siemens Biograph 64-slice PET/CT scanner (Siemens Healthineers, Knoxville, TN), 60 minutes after intravenous injection of 3 MBq⋅kg [^18^F]FDG. Follow-up [^18^F]NaF PET/CT images were obtained on a Siemens Vision scanner (Siemens Healthineers, Erlangen, Germany), 90 minutes after an intravenous injection of 2.0 MBq⋅kg [^18^F]NaF (maximum dosage: 200 MBq). A baseline [^18^F]FDG and follow-up [^18^F]NaF PET/LD CT of one participant with abdominal aorta tracer uptake is shown in Figure 1. Participants were instructed to fast overnight for at least 8 hours and drink 1 L water 1-3 hours before and 0.5 L water after injection of the radiopharmaceutical. Before PET imaging started, a continuous breathing LD CT (80-120 kV, 20-35 mAs, and 5 mm slice thickness) was performed for visualization of anatomical structures and used as attenuation correction map. PET acquisitions were obtained with 2-3 minutes per bed position in 3D setting. Images were reconstructed according to the European Association of Nuclear Medicine guidelines,^13^ using a time of flight iterative reconstruction method (3 iterations, 21 subsets, and voxel-size 3.18 × 3.18 × 2 mm) with point spread function correction. Images were corrected for random coincidences, scatter and attenuation, and were smoothed with a Gaussian filter of 6.5 mm in full width at half maximum.

### Image Analysis

The method of systemic arterial tracer PET activity analysis used in this study, was performed as described previously.^5^,^12^ Briefly, ten arteries were divided into four anatomical segments: carotid arteries (segment 1), ascending aorta and aortic arch (segment 2), descending thoracic and abdominal aorta (segment 3), and iliac and femoral arteries (segment 4). First, the maximal standardized uptake value (SUV_max_) per segment was calculated. Second, by averaging the SUV_max_ of the four individual segments, the _mean_SUV_max_ was calculated. The SUV_max_ values for [^18^F]FDG scans were normalized for fasting pre-scan glucose. Regional arterial target-to-background ratios (_mean_TBR) for both [^18^F]FDG and [^18^F]NaF per segment were calculated by dividing the SUV_max_ by the (in duplex measured) SUV_mean_ derived from the blood pool in the superior caval vein (for segments 1-2) or inferior caval vein (for segments 3-4). _mean_TBR was calculated by averaging the four segments, as measurement for the whole aortic tree for both [^18^F]FDG and [^18^F]NaF. In addition to the per-patient association, a regional analysis between tracers was analyzed including 4 segments in 10 patients, resulting in a comparison of 40 segments. Analyses of [^18^F]NaF and [^18^F]FDG were separated by time and [^18^F]NaF scans were blinded and coded by study number and scan date.

### Arterial Calcification

Based on LD CT-scans, arterial macro-calcification was quantified visually. According to the method of Rominger et al.,^4^ a visual score was assigned for the above mentioned ten arteries. Presence of macro-calcification was scored as 0 (no visual calcification), 1 (plaque covered < 10% of vessel circumference), 2 (plaque covered 10%-25% of vessel circumference), 3 (plaque covered 25%-50% of vessel circumference), or 4 (plaque covered > 50% of vessel circumference). For the calcified plaque (CP) score, the sum of the plaque scores of ten arterial segments was calculated and further in the analysis and manuscript addressed as CPscore.

### Statistical Analysis

Discrete variables are presented as numbers with percentages. Quantitative variables with a normal distribution are presented as median with interquartile range (IQR). Univariate associations were investigated with Spearman’s correlation coefficient (*R*). Wilcoxon’s matched signed rank test was used to asses differences between baseline and follow-up visit. CT-assessed macro-calcification and PWV changes between baseline and follow-up visit were calculated absolutely as [value follow-up]-[value at baseline] and expressed as Delta’s (Δ), relative changes were calculated as ([value follow-up]-[value at baseline])/[value at baseline] and expressed as %change. All statistical analyses were performed with IBM Statistical Package for Social Sciences (SPSS) version 23. *P* < .05 was considered statistically significant.

## Results

Ten patients (seven males, three females), with a median age of 63 [59-69] years at baseline, participated in this substudy. Patient characteristics at baseline and follow-up visit are presented in Table 1 and did not differ significantly from the baseline characteristics of the entire RELEASE cohort.^5^,^12^ HbA1_c_ increased significantly over five years (46 [42-48] vs 52 [44-56] mmol⋅mol (*P* = .012). After five-year follow-up, CPscore had increased significantly (16.0 [5.0-19.5] vs 20 [7.5-24.5] (*P* = .007), with a CPscore %change of 24% [19-33], whereas PWV remained stable (7.98 [6.95-9.29] vs 8.65 [7.79-9.70] m⋅s (*P* = .203), with a PWV %change of 7.2% [− 9.0 to 27] as did other parameters (SBP, BMI, lipids, C-reactive protein, and kidney function (estimated glomerular filtration rate, albumin-creatinin ratio)). Baseline age correlated significantly with _mean_TBR[^18^F]NaF and PWV (*r* = 0.746, *P* = .013, and *r* = 0.654, *P* = .040, respectively).

**Table 1:** Characteristics at baseline and at five-year follow-up

N = 10	Baseline	Five-year follow-up	*P* value
Age (years)	63 [59–69]	69 [63–73]	
Male (%)	70%	70%	
Statin use (%)	60%	70%	
BMI (kg⋅m^2^)	31 [27–36]	31 [28–34]	.799
SBP (mmHg)	138 [127–149]	138 [127–149]	.953
HbA1_c_ (mmol⋅mol)	46 [42–48]	52 [44–56]	**.012**
Total cholesterol (mmol⋅L)	4.85 [4.15–5.33]	4.15 [3.68–5.10]	.106
LDL-cholesterol (mmol⋅L)	3.00 [2.55–3.90]	2.70 [2.35–3.80]	.476
HDL-cholesterol (mmol⋅L)	1.20 [1.10–1.23]	1.10 [0.98–1.33]	.168
Triglycerides (mmol⋅L)	1.45 [1.11–1.99]	1.49 [1.35–1.69]	.760
C-reactive protein (mg⋅L)	1.15 [0.78–2.40]	1.15 [0.83–2.03]	.812
Estimated glomerular filtration rate (ml⋅min*1.73m^2^)	85 [80–101]	87 [84–93]	.878
Albumin-creatinin ratio (mg⋅mmol)	0.45 [0.00–0.80]	0.35 [0.25–0.75]	.646
PWV (m⋅s)	7.98 [6.95–9.29]	8.65 [7.79–9.70]	.203
CT-assessed arterial calcified plaque score	16.0 [5.0–19.5]	20.0 [7.5–24.5]	**.007**
Arterial [^18^F]-FDG uptake (_mean_TBR)	2.12 [1.82–2.49]		
Arterial [^18^F]-NaF uptake (_mean_TBR)		2.20 [2.03–2.97]	

### Predictive Value of PET and PWV-Assessed Arterial Disease

**Figure 2:**
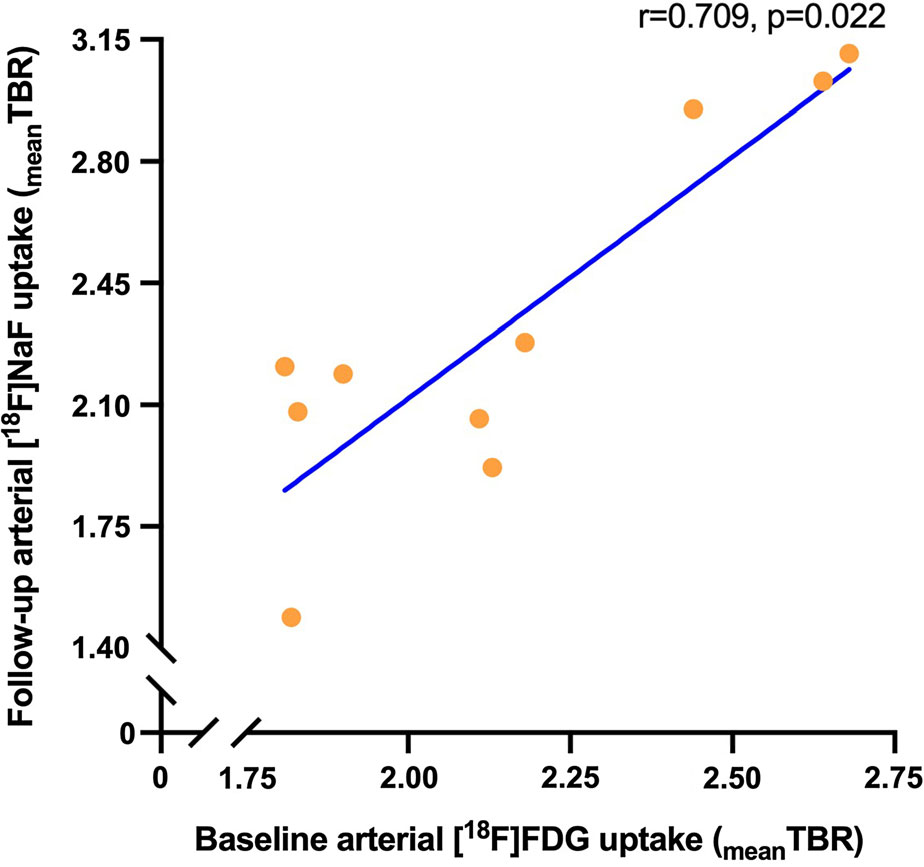
Baseline systemic arterial inflammation is related to five-year follow-up systemic arterial micro-calcification. Correlation between arterial [^18^F]-fluordeoxyglucose ([^18^F]FDG) uptake at baseline and arterial [^18^F]-sodium fluoride ([^18^F]NaF) uptake at five-year follow-up, expressed as target-to-background ratio (TBR). TBR was calculated by dividing the maximal standardized uptake value (SUVmax) of the arteries by the mean standardized uptake value (SUVmean) of the caval veins (blood pool)

**Figure 3:**
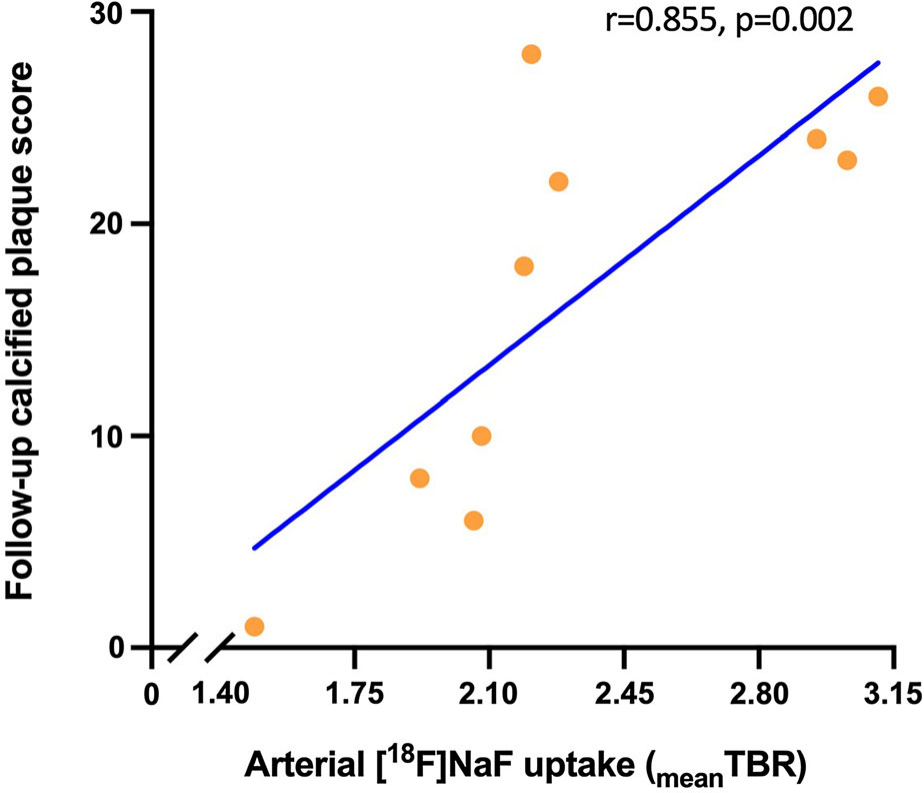
Systemic arterial micro-calcification was related to systemic arterial macro-calcification over five years. Correlation between systemic positron emission tomography-assessed arterial [^18^F]-sodium fluoride ([^18^F]NaF) uptake (expressed as _mean_target to background ratio (TBR), calculated by averaging ten TBRs (maximal standardized uptake value (SUVmax) divided by the mean standardized uptake value (SUVmean) of the blood pool)) and follow-up low dose computed tomography-assessed arterial macro-calcification (expressed as calcified plaque score, measured in whole aortic tree^4^)

Baseline _mean_TBR[^18^F]FDG demonstrated a strong positive correlation with five-year follow-up _mean_TBR[^18^F]NaF (*r* = 0.709, *P* = .022, Figure 2). Also, the regional association between [^18^F]FDG and [^18^F]NaF showed a significant correlation over five years (*r* = 0.523, *P* = .001). Baseline _mean_TBR[^18^F]FDG did not correlate with age (*r* = 0.306, *P* = .390), baseline CPscore (*r* = 0.334, *P* = .345), follow-up CPscore (*r* = 0.345, *P* = .328), follow-up PWV (*r* = − 0.103, *P* = .777), ΔCPscore (*r* = 0.389, *P* = .267) or ΔPWV (*r* = − 0.442, *P* = .200). CPscore at baseline and follow-up were strongly correlated (*r* = 0.960, *P* < .001). Follow-up _mean_TBR[^18^F]NaF did correlate with ΔCPscore and CPscore at baseline and follow-up (*r* = 0.735, *P* = .016, *r* = 0.845, *P* = .002 and *P* = 0.855, *P* = .002 (Figure 3), respectively), while it was not associated with CPscore %change (*r* = − 0.152, *P* = .675), PWV %change (*r* = − 0.406, *P* = .244) and ΔPWV (*r* = − 0.285, *P* = .425). Furthermore, follow-up _mean_TBR[^18^F]NaF showed a trend toward a significant positive association with baseline PWV (*r* = 0.588, *P* = .074) but not with follow-up PWV (*r* = − 0.018, *P* = .960). Also, baseline and follow-up PWV did not correlate with CPscore at baseline (*r* = 0.377, *P* = .283 and *r* = 0.079, *P* = .828, respectively) or at follow-up (*r* = 0.527, *P* = .117 and *r* = 0.285, *P* = .425, respectively).

## Discussion

In this five-year follow-up study, baseline arterial [^18^F]FDG uptake correlated positively with follow-up arterial [^18^F]NaF uptake as interrelated molecular imaging markers of development of systemic arterial disease in patients with early T2DM without overt cardiovascular disease. Arterial [^18^F]NaF uptake demonstrated a significant positive correlation with the CT-assessed whole aortic tree macro-calcification burden. After five years of follow-up we observed that, compared with baseline, CPscore increased significantly, while PWV did not. This suggests that, without significantly affecting arterial stiffness, systemic arterial disease increased in five years in patients with early T2DM and that the development of atherosclerosis seems to progress from systemic arterial inflammation to systemic arterial micro-calcification over time.

Although our cohort has a limited sample size, we believe these observations add significance to the understanding of arterial [^18^F]FDG uptake in T2DM. To the best of our knowledge, no similar follow-up studies with arterial [^18^F]FDG and [^18^F]NaF uptake have been reported in patients with T2DM. However, previous studies did investigate the relation between arterial [^18^F]FDG and [^18^F]NaF uptake at the same time. Derlin et al. observed *in vivo* colocalized uptake of both [^18^F]FDG and [^18^F]NaF in only 6.5% of focal arterial lesions in carotid arteries and aorta.^14^ This limited relation between [^18^F]FDG and [^18^F]NaF uptake might be explained by the fact that PET images were performed simultaneously after separate administration while these tracers are markers of a different stage of development of atherosclerosis.^3^ An additional import distinction is that the current study focused on the whole aortic tree, reflecting a more systemic measurement approach of arterial tracer uptake. This is important, since atherosclerosis is generalized arterial disease and not limited to specific sites. A similar approach was used in patients with pseudoxanthoma elasticum, in which a relation between arterial [^18^F]FDG and [^18^F]NaF uptake was not observed.^9^ In this latter study, PET scans were performed within a very short time interval of just a few days. The observation that arterial [^18^F]FDG and [^18^F]NaF uptake do not colocalize in parallel PET scans emphasizes that these tracers reflect a different stage of arterial disease and appear not to coincide in an arterial lesion.^3^ By demonstrating a correlation of arterial [^18^F]FDG with [^18^F]NaF uptake over a time frame of five years, the current study sheds light on the potential temporal arterial disease progression from arterial inflammation to arterial micro-calcification, but not macro-calcification. This accentuates the importance of inflammation as risk factor in the development of atherosclerotic diseases.^1^ In contrast to our brief report results, other studies did show a correlation between arterial [^18^F]FDG and macro-calcification, maybe due to low power in the current study.^15^

Our data suggest that micro-calcifications appear related to the retrospective and concurrently measured burden of arterial macro-calcification, but not with the relative change in calcification over five years. Few clinical studies have been performed on aortic [^18^F]NaF uptake in relation to arterial disease progression. We and others previously demonstrated that focal [^18^F]NaF uptake is a reliable marker to identify active atherosclerotic calcification formation.^16^,^17^ In these studies it was observed that calcification patterns of [^18^F]NaF and CT are different, revealing a different stage of the calcification process. Next to that, in patients with aortic stenosis it was demonstrated that baseline aortic valve [^18^F]NaF uptake correlated with the progression of calcium content after one year.^18^ This result is in line with our current study, in which systemically assessed arterial [^18^F]NaF activity was related to the five-year increase of macrocalcification, highlighting [^18^F]NaF as a marker of active calcium buildup. In contrast, in a study among postmenopausal women, in which a [^18^F]NaF PET scan was performed for the assessment of bone mineralization, baseline aortic [^18^F]NaF uptake did not predict aortic calcification and its four-year progression.^19^ However, the authors had adequately addressed that they only included women without cardiovascular risk factors and that results may not be directly generalizable to men or other (high risk) populations, which we particularly included in our study. With the current study we underline previous conclusions that systemic arterial [^18^F]NaF uptake reveals arterial macro-calcification in high risk patients with T2DM. Although speculatively, these data underline that atherosclerotic progression already takes place at an early stage of T2DM and progression from arterial inflammation into micro-calcification on a systemic level already occurs in five years.

This long-term follow-up study also has some limitations. First, our proof-of-concept study is limited by the small sample size, resulting in an inability to adjust for additional parameters (such as sex and drug use) which potentially could have influenced our outcomes. Second, we could not assess changes over time in arterial [^18^F]FDG and [^18^F]NaF uptake because no [^18^F]NaF PET scan was performed at baseline and no [^18^F]FDG PET scan was repeated at the follow-up visit. Third, while [^18^F]NaF uptake was related to the preceding and concurrent macro-calcification cross-sectionally we did not study the role of [^18^F]NaF uptake in predicting macrocalcification progression since baseline [^18^F]NaF PET scans was not performed. Finally, more advanced methods of PET image analyses were developed in the previous years between our baseline and follow-up analyses. For instance, blood pool measurements in the right atrium seem more reproducible and quantification of aortic whole vessel PET tracer uptake has improved.^20^ However, even with this small sample size, we already observed correlations of systemic arterial [^18^F]NaF uptake with the active process of the arterial disease progression with molecular imaging. These results prompts to perform similar analyses in larger cohorts.

## Conclusion

In this study it was demonstrated for the first time that, in patients with early T2DM without overt cardiovascular disease, systemic arterial inflammation is prospectively related to systemic arterial micro-calcification after five-year follow-up, as measured with arterial [^18^F]FDG and [^18^F]NaF uptake, respectively. This implicates that arterial disease is present in an early phase of T2DM and that this arterial disease leads to a significant increase of arterial calcification. Although larger cohort studies are needed to investigate whether these changes on PET are related to T2DM parameters and are predictive for clinical outcomes, we believe that the current study contributes to the current understanding of the temporal interrelation of these tracers in arterial disease.

## New Knowledge Gained

In cardiovascular high risk patients with type 2 diabetes mellitus, PET-assessed systemic arterial [^18^F]FDG uptake is positively and prospectively associated with five-year follow-up systemic arterial [^18^F]NaF uptake.

## Supplementary Information

Below is the link to the electronic supplementary material.

Supplementary file1 (PPTX 590 kb)

## Abbreviations

[^18^F]FDG: [^18^F]-fluordeoxyglucose
[^18^F]NaF: [^18^F]-sodium fluoride
CP: Calcium plaque
CT: Computed tomography
LD: Low dose
PET: Positron emission tomography
PWV: Pulse wave velocity
SUV: Standard uptake value
T2DM: Type 2 diabetes mellitus
TBR: Target to background ratio
