# Supplementary material for: [18F]FDG and [18F]NaF as PET markers of systemic atherosclerosis progression: A longitudinal descriptive imaging study in patients with type 2 diabetes mellitus
Source: J Nucl Cardiol. 2021 Sep 13;29(4):1702–9. doi: 10.1007/s12350-021-02781-w (PMC9345832; doi:10.1007/s12350-021-02781-w)
Supplement: Supplementary file 1 — Supplementary file1 (PPTX 590 kb) [file 12350_2021_2781_MOESM1_ESM.pptx]

## Slide 1
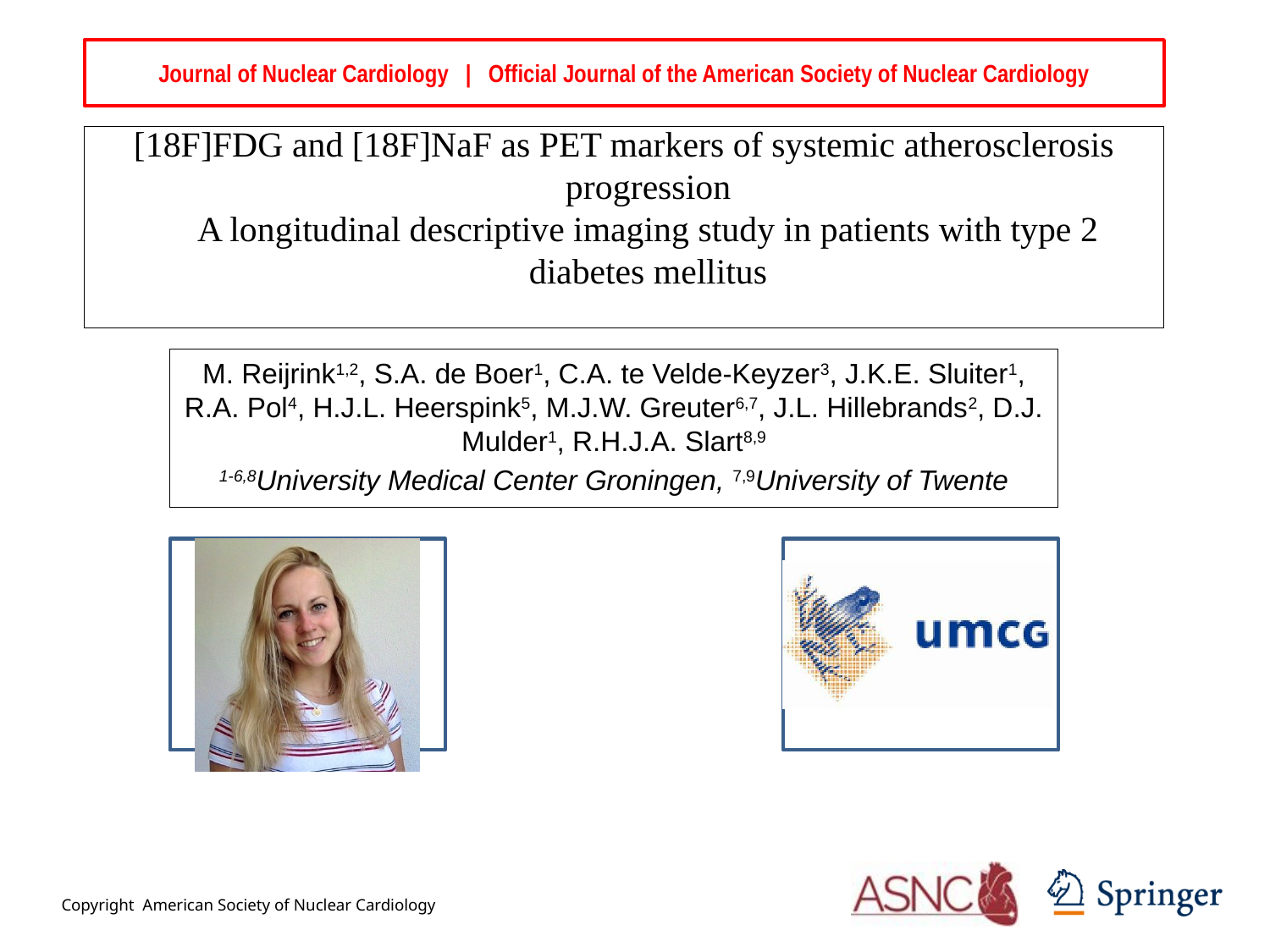

Journal of Nuclear Cardiology | Official Journal of the American Society of Nuclear Cardiology
# [18F]FDG and [18F]NaF as PET markers of systemic atherosclerosis progression A longitudinal descriptive imaging study in patients with type 2 diabetes mellitus
M. Reijrink1,2, S.A. de Boer1, C.A. te Velde-Keyzer3, J.K.E. Sluiter1, R.A. Pol4, H.J.L. Heerspink5, M.J.W. Greuter6,7, J.L. Hillebrands2, D.J. Mulder1, R.H.J.A. Slart8,9
1-6,8University Medical Center Groningen, 7,9University of Twente
Head shot of author
required
Optional
Copyright American Society of Nuclear Cardiology

## Slide 2
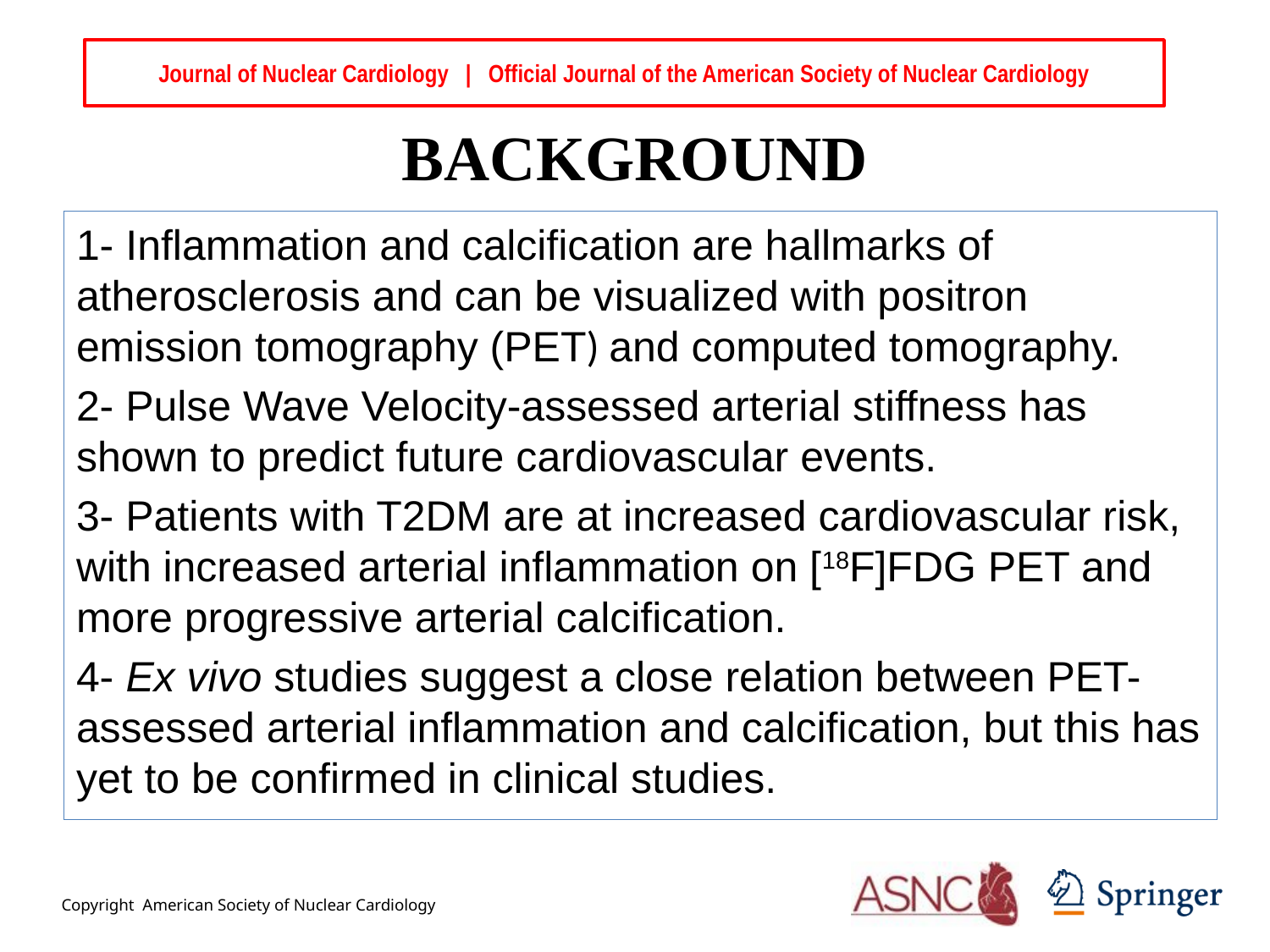

Journal of Nuclear Cardiology | Official Journal of the American Society of Nuclear Cardiology
# BACKGROUND
1- Inflammation and calcification are hallmarks of atherosclerosis and can be visualized with positron emission tomography (PET) and computed tomography.
2- Pulse Wave Velocity-assessed arterial stiffness has shown to predict future cardiovascular events.
3- Patients with T2DM are at increased cardiovascular risk, with increased arterial inflammation on [18F]FDG PET and more progressive arterial calcification.
4- Ex vivo studies suggest a close relation between PET-assessed arterial inflammation and calcification, but this has yet to be confirmed in clinical studies.
Copyright American Society of Nuclear Cardiology

## Slide 3
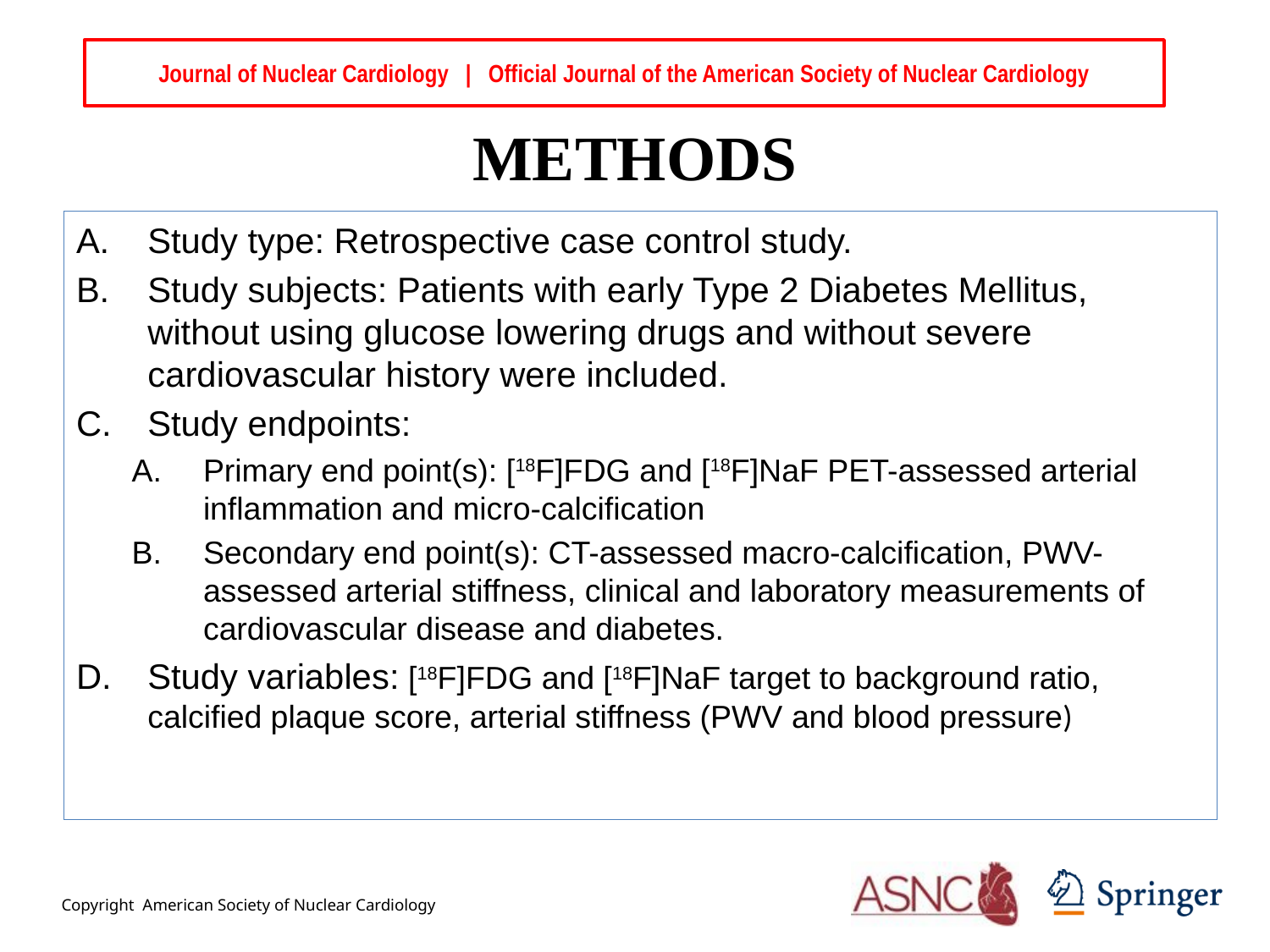

Journal of Nuclear Cardiology | Official Journal of the American Society of Nuclear Cardiology
# METHODS
Study type: Retrospective case control study.
Study subjects: Patients with early Type 2 Diabetes Mellitus, without using glucose lowering drugs and without severe cardiovascular history were included.
Study endpoints:
Primary end point(s): [18F]FDG and [18F]NaF PET-assessed arterial inflammation and micro-calcification
Secondary end point(s): CT-assessed macro-calcification, PWV-assessed arterial stiffness, clinical and laboratory measurements of cardiovascular disease and diabetes.
Study variables: [18F]FDG and [18F]NaF target to background ratio, calcified plaque score, arterial stiffness (PWV and blood pressure)
Copyright American Society of Nuclear Cardiology

## Slide 4
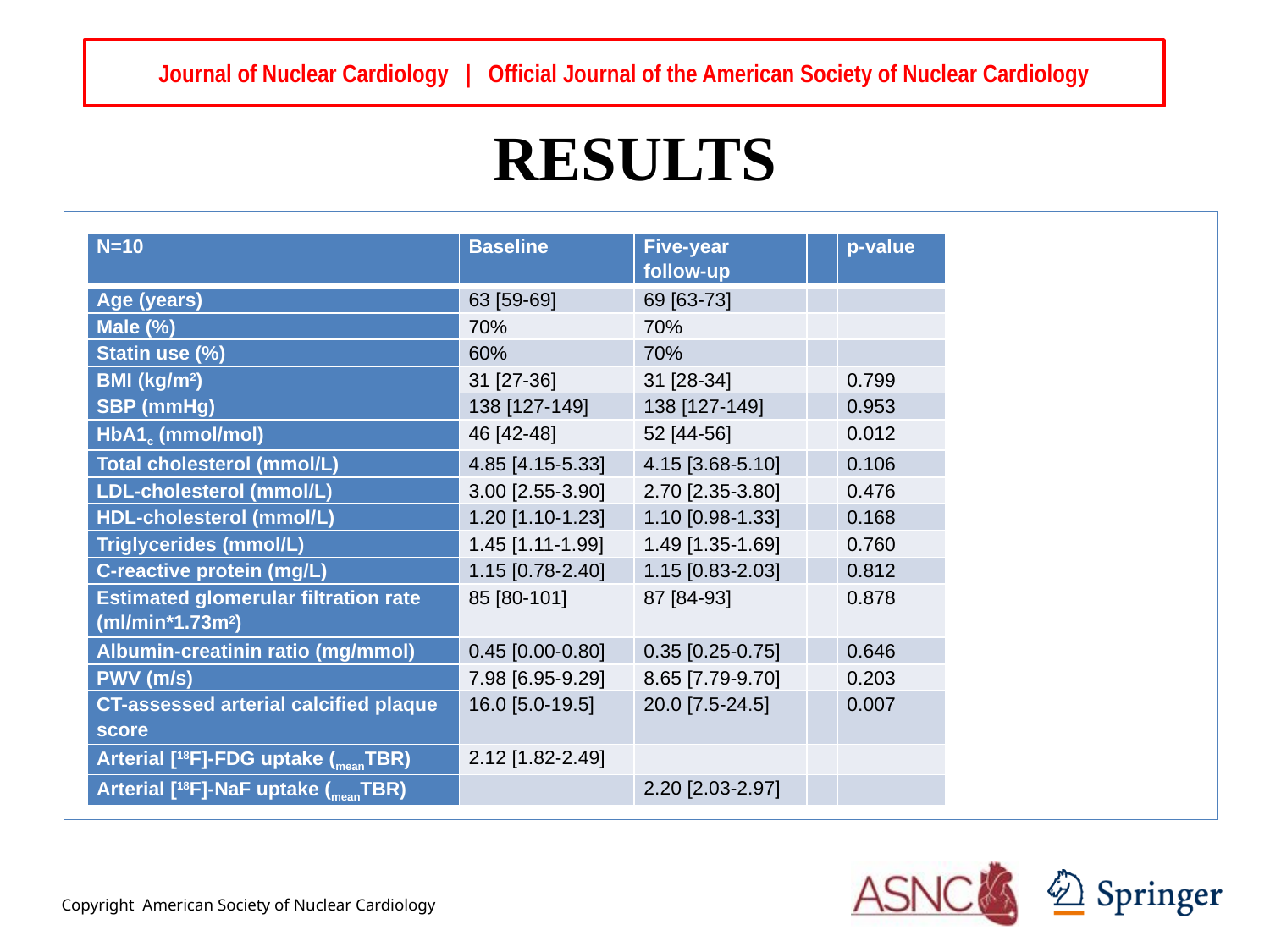

Journal of Nuclear Cardiology | Official Journal of the American Society of Nuclear Cardiology
# RESULTS
In
If figure, insert legend
| N=10 | Baseline | Five-year follow-up | | p-value |
| --- | --- | --- | --- | --- |
| Age (years) | 63 [59-69] | 69 [63-73] | | |
| Male (%) | 70% | 70% | | |
| Statin use (%) | 60% | 70% | | |
| BMI (kg/m2) | 31 [27-36] | 31 [28-34] | | 0.799 |
| SBP (mmHg) | 138 [127-149] | 138 [127-149] | | 0.953 |
| HbA1c (mmol/mol) | 46 [42-48] | 52 [44-56] | | 0.012 |
| Total cholesterol (mmol/L) | 4.85 [4.15-5.33] | 4.15 [3.68-5.10] | | 0.106 |
| LDL-cholesterol (mmol/L) | 3.00 [2.55-3.90] | 2.70 [2.35-3.80] | | 0.476 |
| HDL-cholesterol (mmol/L) | 1.20 [1.10-1.23] | 1.10 [0.98-1.33] | | 0.168 |
| Triglycerides (mmol/L) | 1.45 [1.11-1.99] | 1.49 [1.35-1.69] | | 0.760 |
| C-reactive protein (mg/L) | 1.15 [0.78-2.40] | 1.15 [0.83-2.03] | | 0.812 |
| Estimated glomerular filtration rate (ml/min\*1.73m2) | 85 [80-101] | 87 [84-93] | | 0.878 |
| Albumin-creatinin ratio (mg/mmol) | 0.45 [0.00-0.80] | 0.35 [0.25-0.75] | | 0.646 |
| PWV (m/s) | 7.98 [6.95-9.29] | 8.65 [7.79-9.70] | | 0.203 |
| CT-assessed arterial calcified plaque score | 16.0 [5.0-19.5] | 20.0 [7.5-24.5] | | 0.007 |
| Arterial [18F]-FDG uptake (meanTBR) | 2.12 [1.82-2.49] | | | |
| Arterial [18F]-NaF uptake (meanTBR) | | 2.20 [2.03-2.97] | | |
Copyright American Society of Nuclear Cardiology

## Slide 5
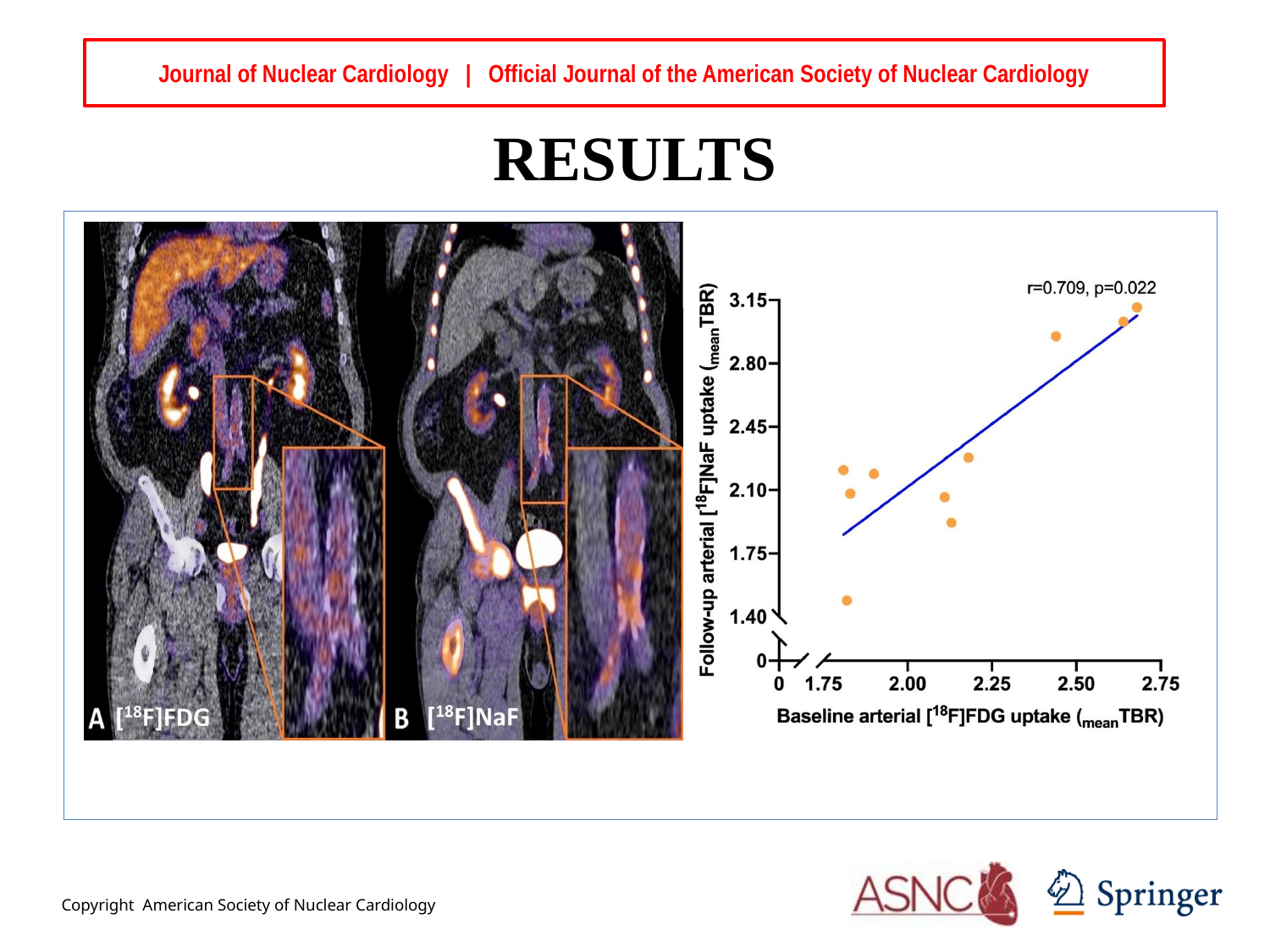

Journal of Nuclear Cardiology | Official Journal of the American Society of Nuclear Cardiology
# RESULTS
Copyright American Society of Nuclear Cardiology

## Slide 6
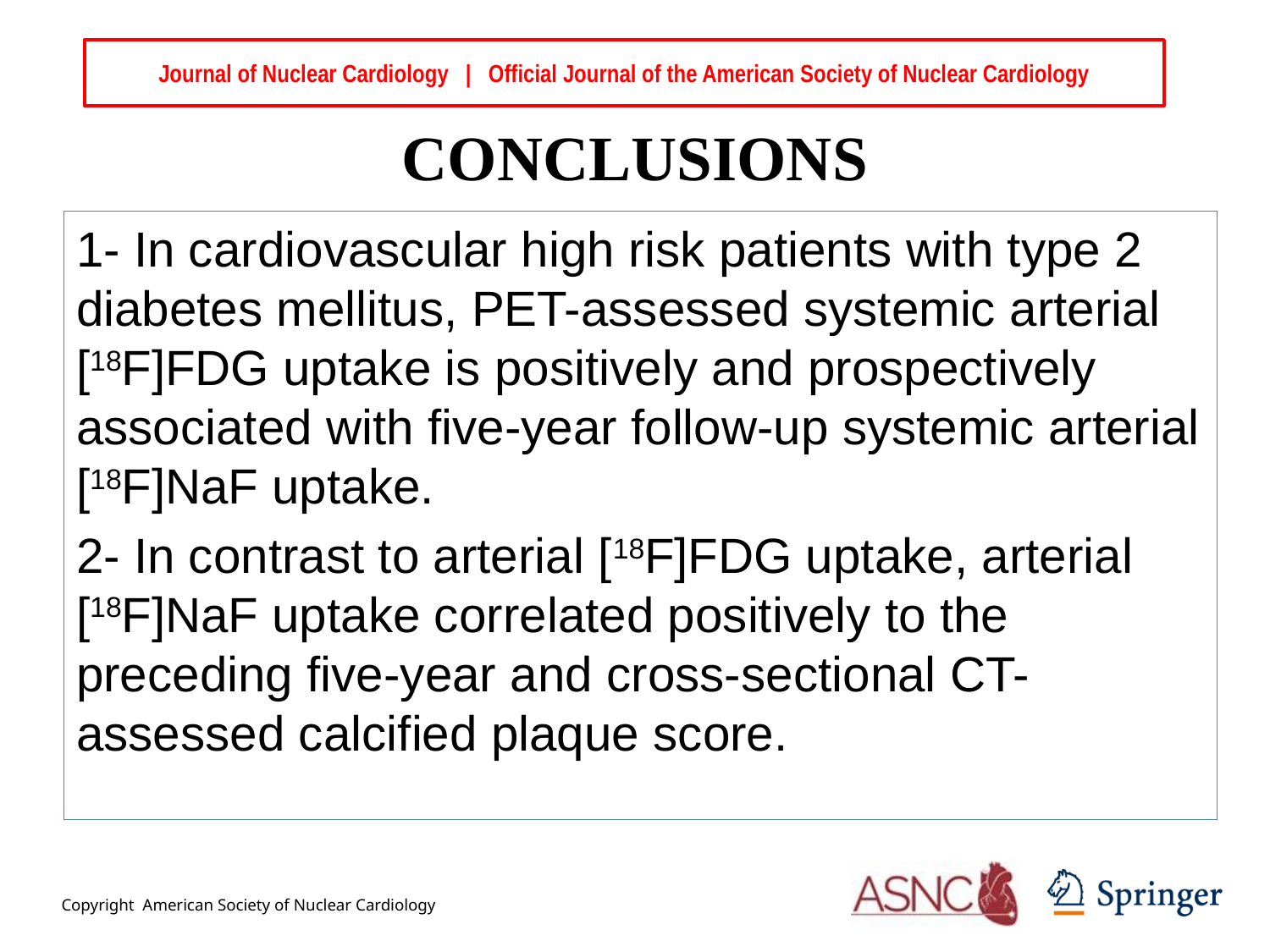

Journal of Nuclear Cardiology | Official Journal of the American Society of Nuclear Cardiology
# CONCLUSIONS
1- In cardiovascular high risk patients with type 2 diabetes mellitus, PET-assessed systemic arterial [18F]FDG uptake is positively and prospectively associated with five-year follow-up systemic arterial [18F]NaF uptake.
2- In contrast to arterial [18F]FDG uptake, arterial [18F]NaF uptake correlated positively to the preceding five-year and cross-sectional CT-assessed calcified plaque score.
Copyright American Society of Nuclear Cardiology
